# The Association Between Family Health and Frailty With the Mediation Role of Health Literacy and Health Behavior Among Older Adults in China: Nationwide Cross-Sectional Study

**DOI:** 10.2196/44486

**Published:** 2023-06-27

**Authors:** Haomiao Li, Yibo Wu, Zhongliang Bai, Xiwu Xu, Dai Su, Jiangyun Chen, Ruibo He, Ju Sun

**Affiliations:** 1 School of Political Science and Public Administration Wuhan University Wuhan China; 2 School of Public Health Peking University Beijing China; 3 School of Health Services Management Anhui Medical University Hefei China; 4 Faculty of Education, Health and Wellbeing, University of Wolverhampton Wolverhampton United Kingdom; 5 Beijing Hospital Beijing China; 6 Department of Health Management and Policy School of Public Health Capital Medical University Beijing China; 7 Institute of Health Management Southern Medical University Guangzhou China; 8 College of Finance and Public Administration, Hubei University of Economics Wuhan China

**Keywords:** family health, frailty, health literacy, health behavior, healthy aging

## Abstract

**Background:**

Family health develops from the intersection of the health of each family member and their interactions and capacities as well as the family’s internal and external resources. Frailty is the most prominent and typical clinical manifestation during population aging. Family health may be effective in addressing frailty, and this association may be mediated by health literacy and health behaviors. Until now, it is unclear whether and how family health affects frailty in older adults.

**Objective:**

This study aimed to examine the associations between family health and frailty and the mediation roles of health literacy and health behaviors.

**Methods:**

A total of 3758 participants aged ≥60 years were recruited from a national survey conducted in 2022 in China for this cross-sectional study. Family health was measured using the Short Form of the Family Health Scale. Frailty was measured using the Fatigue, Resistance, Ambulation, Illnesses, and Loss of weight (FRAIL) scale. Potential mediators included health literacy and health behaviors (not smoking, not having alcohol intake, physical exercise for ≥150 minutes per week, longer sleep duration, and having breakfast every day). Ordered logistic regression was applied to explore the association between family health and frailty status. Mediation analysis based on Sobel tests was used to analyze the indirect effects mediated by health literacy and behaviors, and the Karlson-Holm-Breen method was used to composite the indirect effects.

**Results:**

Ordered logistic regression showed that family health is negatively associated with frailty (odds ratio 0.94, 95% CI 0.93-0.96) with covariates and potential mediators controlled. This association was mediated by health literacy (8.04%), not smoking (1.96%), longer sleep duration (5.74%), and having breakfast every day (10.98%) through the Karlson-Holm-Breen composition.

**Conclusions:**

Family health can be an important intervention target that appears to be negatively linked to frailty in Chinese older adults. Improving family health can be effective in promoting healthier lifestyles; improving health literacy; and delaying, managing, and reversing frailty.

## Introduction

### Background

The rapid aging of populations will lead to a greater chronic disease burden on the whole society [1]. Diseases such as hypertension, diabetes, cancer, stroke, arthritis, dementia, and related disease burdens will continue to increase [2]. From the perspective of disease occurrence and development, the immune functions of older adults gradually decrease with age, their susceptibility to adverse stimuli increases, and disease incidence rates will inevitably increase. However, there are substantially individual differences in the occurrence, development, prognosis, and progression of diseases and the consequent quality of life. These differences are mainly due to frailty [3,4]. *Frailty* refers to a homeostatic imbalance in the body, between unhealthy and serious damaging status due to a decline in physical, psychological, or cognitive function. It is the most prominent and typical clinical manifestation during population aging [5]. In addition, “prefrailty,” which is defined as a complex multifactorial and multidimensional state associated with physiological and deleterious processes that develop over time, is an intermediate stage in the progression from robust to frailty [6]. Compared with frailty, prefrailty has a higher prevalence but a lower level of damage to older adults. In addition, prefrailty is more clinically reversible [7,8], which highlights the importance of early identification and intervention to reverse it or prevent the progression to frailty. Previous studies have shown that both prefrailty and frailty are associated with an increased risk of hospitalization, functional decline, progression to long-term care, and death [9]. Delaying the occurrence of prefrailty or frailty and promoting their reversal can play a substantial role in the promotion of healthy aging.

Family-oriented health promotions are promising and effective strategies because the family unit is both a resource and a priority group that needs preventive and curative services across its members’ life course [10]. In particular, “family health” as a relatively new concept is receiving more attention from scholars. Weiss-Laxer et al [11] defined family health as a resource at the family unit level that develops from the intersection of the health of each family member and their interactions and capacities with the family’s physical, social, emotional, economic, and medical resources. Family health integrates the key elements of the previous concepts of family structure, family function, and family social network; seeks to strengthen the ability of families to obtain external resources and sociality; and emphasizes health-related elements, linking individual health with social health. Therefore, family health may be an important target for health intervention. Regarding strategies for preventing and controlling frailty, researchers have yet to determine whether interventions through family health are effective.

Health literacy and health behaviors may be effective pathways to address the association between family health and frailty. Health literacy is defined as a cognitive and social skill that determines an individual’s motivation and ability to access, understand, and use information in a way that promotes and maintains health. Low health literacy is associated with a poor understanding of one’s medical condition, poor self-care, delayed care seeking, and lower use of preventive services; it can also affect disease management and outcomes in patients with chronic conditions [12,13]. Adequate health literacy can prevent frailty in older adults and plays a positive role in intervention and the management of frail, community-dwelling older adults [14,15]. Residents’ health literacy may be affected by family structure, income level, information delivery, and other family members’ education levels. Behaviors are the most concrete, visible aspect of family functioning. Family structure, processes, and cognitions are expressed through family behaviors [16]. There are a range of mechanisms underlying relationships between family and health behaviors, including promoting health-seeking or health treatment behaviors providing access, opportunities, and resources for a range of health behaviors [17]. Previous studies have also demonstrated the relationship between healthy behaviors, such as diet and exercise, and frailty [5,18]. Nevertheless, the associations between family health, health literacy, and health behaviors and frailty have not been explored.

### Objective and Hypotheses

China is one of the countries with the fastest aging population and faces unprecedented challenges in the face of such a rapid aging process. Therefore, frailty prevention and management is essential and urgent in China, and family health may be an effective subject for intervention. This study explored whether and how family health could decrease the frailty risk in older populations. We proposed two hypotheses: (1) family health is positively associated with the prevalence of prefrailty and frailty and (2) the association between family health and frailty status is mediated by health literacy and health behaviors.

## Methods

### Sampling and Participants

The data are from a national survey, conducted from June 20, 2022, to August 31, 2022, in 148 cities; 202 districts and counties; 390 townships, towns, or streets; 780 communities or villages (excluding Hong Kong, Macao, and Taiwan) from 23 provinces; 5 autonomous regions; and 4 municipalities directly under the central government in China, initiated by the Peking University School of Public Health. The sampling ratio was determined based on the population proportion provided by the seventh national census data. At least 500, 1000, 1500, 2000, or 2500 individuals were sampled from each province, autonomous region, or municipality directly under the central government. The sample size was estimated to be 20,000. Finally, in the municipal, district, county, township or town, street, and community level, individual were sampled according to quota attribute, including sex and age.

To conduct the survey, the investigator set up a questionnaire survey site in the health service center or a relevant health service station of the sampling community in charge and then posted a poster and issued a paper or electronic invitation to recruit respondents. The investigator checked the identities of all respondents, solicited their informed consent, and determined whether the respondents met the inclusion criteria for the study participants. The targeted participants had to be aged >12 years, able to understand each item of the questionnaire, and able to complete the questionnaire on their own or with the help of an investigator. People who were confused, were experiencing mental health difficulties or cognitive impairment, or were unwilling to participate in the survey were excluded. All the participants voluntarily participated in the study and signed an informed consent form. In total, 21,916 questionnaires were collected after quota sampling. The survey protocol has been published [19].

**Figure 1:**
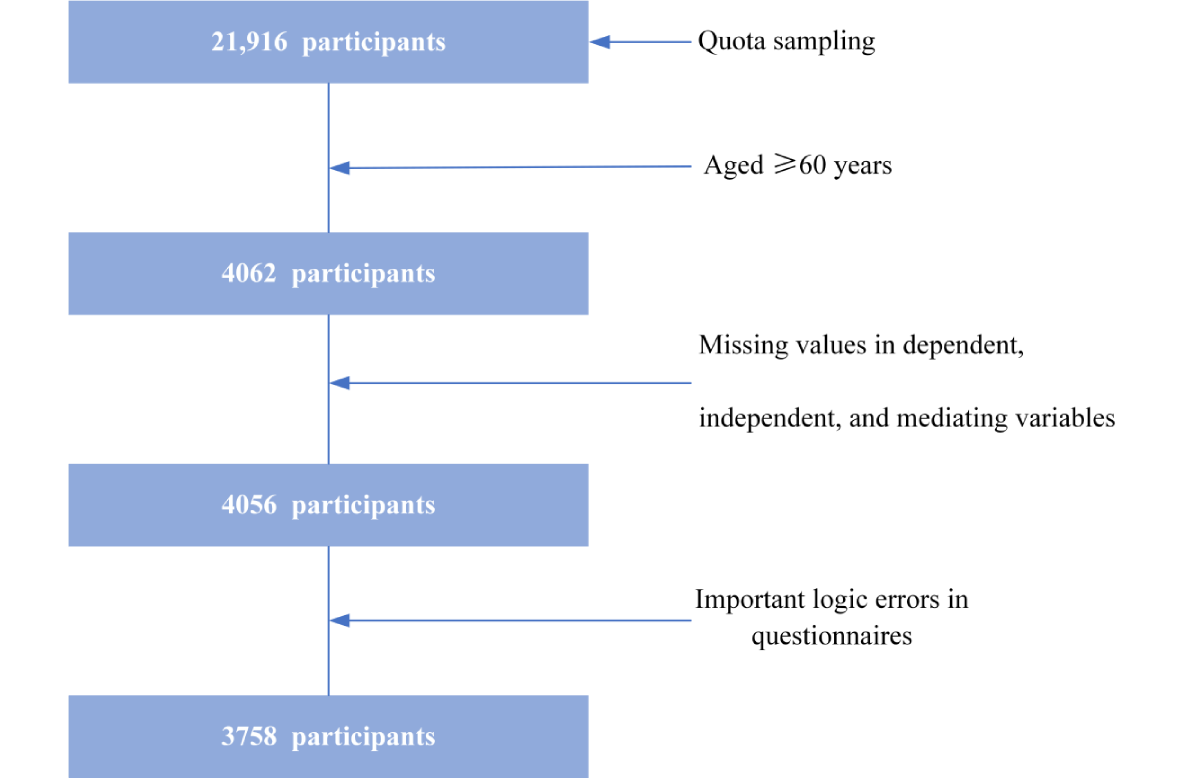
Sample selection process for this cross-sectional study.

In this study, the data of participants aged <60 years; those whose questionnaires did not include details on the independent, dependent, and potential mediating variables; and those whose questionnaires had logical errors (mainly contradictions and discrepancies between important variables such as age, family type, and marital status) were also excluded (details are shown in Figure 1). In total, 3758 participants were included in the final analysis.

### Variables

#### Frailty

Frailty was measured using the Fatigue, Resistance, Ambulation, Illnesses, and Loss of weight (FRAIL) scale [20], which has been validated in older Chinese populations (Multimedia Appendix 1) [21-23]. We assessed the presence of fatigue and loss of body weight by participants responding “yes” to the following items in the self-reported questionnaire: “Were you tired most of last week?” and “Have you experienced an unexplained loss of more than 5% of your body weight in the last year?” The presence of resistance and ambulation problems were assessed by a “yes” answer to the following questions: “Can you go up a staircase?” and “Can you walk a block (500 meters) away?” Illness was assessed based on the total number of chronic diseases that participants had (at least 5 diseases had to be present), and the number was then dichotomized into a binary variable. All the abovementioned 5 variables were coded as 0 (“no”) or 1 (“yes”), with 1 indicating the presence of deficits. The total deficits were summed to calculate a frailty score that ranged from 0 to 5. On the basis of previous studies, participants who scored 0 were defined as robust, those who scored 1 or 2 were defined as prefrail, and those who scored ≥3 were defined as frail [24].

#### Family Health

Family health was measured using the Short Form of the Family Health Scale, which was translated into Chinese with the consent of the original author. The Chinese version of the Short Form of the Family Health Scale has good reliability and validity and can be used to assess the level of family health of Chinese residents [25]. It contains four dimensions: (1) family, social, or emotional health processes; (2) family healthy lifestyle; (3) family health resources; and (4) family external social support. The scores of the items and calculations for family health ranged from 10 to 50, with a higher score indicating better family health (Table 1). Cronbach α of the sample in this study was .634, which was deemed as indicating acceptable reliability, and the Kaiser-Meyer-Olkin value was 0.702, which was deemed as indicating acceptable validity.

**Table 1:** Short Form of the Family Health Scale.

Item number^a^	Dimensions	Items
1	Family, social, or emotional health processes	We support each other.
2	Family, social, or emotional health processes	I feel safe in my family relationships.
3	Family, social, or emotional health processes	We stay hopeful even in difficult times.
4	Family healthy lifestyle	We help each other in seeking health care services when needed (such as making physician’s appointments).
5	Family healthy lifestyle	We help each other make healthy changes.
6	Family health resources	We do not trust doctors and other health professionals.
7	Family health resources	My family did not have enough money at the end of the month after bills were paid.
8	Family health resources	My family did not have adequate housing.
9	Family external social supports	We have people outside of our family we can turn to when we have problems at school or work.
10	Family external social supports	If we needed financial help, we have people outside of our family we could turn to for a loan (eg, for RMB 1000 [US $140]).

^a^Items 1 to 5 and 9 to 10 were positively scored (strongly disagree=1, somewhat disagree=2, neither agree nor disagree=3, somewhat agree=4, and strongly agree=5), and items 6 to 8 were negatively scored (strongly disagree=5, somewhat disagree=4, neither agree nor disagree=3, somewhat agree=2, and strongly agree=1). The total score is the sum of each item, with a score ranging from 10 to 50, with a higher score indicating better family health.

#### Mediators

The mediators measured in this study were health literacy and health behaviors. Health literacy was measured using the new short-form health literacy instrument, which included 9 items (Multimedia Appendix 2). Scores ranged from 9 to 36 points, with a high score indicating higher levels of health literacy. Health behaviors included smoking, alcohol intake, physical exercise, sleep, and diet. Smoking status and alcohol intake were set as binary variables (1=yes and 0=no). Physical exercise was assessed by weekly exercise time, including aggravating activities (power sports, fast running, ball games, aerobics, and fast cycling), moderate-intensity physical activity (handling goods, medium-speed cycling, jogging, and table tennis, excluding walking), and light aerobic exercise (walking for at least 10 min). Exercise time was calculated by summing up all activity time (minute), and ≥150 minutes was deemed as a healthy style (0: <150 minutes and 1: ≥150 minutes) [26]. Sleep behavior was assessed by sleep duration per night (1: <5 hours, 2: 5-6 hours, 3: 6-7 hours, and 4: >7 hours). Diet behavior was measured based on whether the participant had breakfast every day (1=yes and 0=no).

#### Covariates

The covariates were initially identified based on previous studies and general knowledge. Then, the enrolled covariates were selected based on their association with the independent variable and their impact on the change in the association between the independent and dependent variables. Age, sex, and potential mediators were included as fixed covariates to be controlled. Other covariates were included as potential confounders in the final models if they changed the estimates of the effect of family health on frailty status by >10% or were significantly associated with frailty status based on generalized linear regression [27]. The final covariates included age, sex (0=male and 1=female), family type (1=core family, 1=backbone family, 2=joint family, 3=conjugal family, 4=single-parent family, and 5=other), residence (0=urban and 1=rural), marital status (0=married and 1=divorced, widowed, or unmarried), number of children (grouped by 0, 1, 2, and ≥3), public insurance coverage (0=not covered and 1=covered), and BMI and self-rated health (scoring from 0 to 100).

### Statistical Analysis

Sociodemographic characteristics, health literacy, health behaviors, and family health of participants were summarized using frequencies (percentages) or means and SDs, grouped by frailty status. Statistical differences were tested using Kruskal-Wallis one-way analysis for the continuity variables and chi-square tests for the categorical variables.

Ordered logistic regressions were applied to assess the association between family health, including its 4 dimensions; health behaviors; and frailty, with covariates controlled. Odds ratios (ORs) and 95% Cis for the risk of frailty were estimated. A generalized additive model and smoothing curve fitting were further used to address the potential nonlinear relationship between family health and the risk of frailty, with potential mediators and covariates controlled. The generalized additive model allows us to fit the model using a nonlinear smoothing term without prior knowledge of the relationship between the dependent and independent variables, which allows the association to be visualized more intuitively [28].

Sobel tests were then applied to measure the significance of the mediating effects of health literacy and health behaviors. To assess the effect sizes of the mediators, Karlson-Holm-Breen (KHB) methods were used to composite the indirect effects with the ordered logistic model. For health behaviors, the healthier groups were used as references.

The significance level was set at a *P* value of <.05 for all the hypothesis tests. Data were analyzed using Stata (version 17.0; StataCorp) [29] and R (version 3.6.3; R Foundation for Statistical Computing).

### Sensitivity and Subgroup Analysis

Several sensitivity analyses were conducted to validate the results. First, as the prevalence of frailty was relatively low in this study, we set the absolute value of the frailty score (0-5 points) as the dependent variable and repeated the KHB decomposition methods. As the frailty score followed a Poisson distribution, we applied negative binomial regression as the model for analysis during the KHB decomposition. Second, in the questionnaire, physical exercise included some high-intensity exercises, which may not be universal or normal among older populations. Therefore, we used the number of days walking (at least 10 minutes) per week instead of physical exercise time. Third, the optimal sleep duration for older adults was not consistent with previous studies. Therefore, we used self-rated sleep quality (0=*very bad*, 1=*bad*, 2=*good*, and 3=*very good*) instead of sleep duration.

Furthermore, the definition of “older adults” was varied in recent studies. The participants in this study were adults aged ≥60 years, which was consistent with some previous studies and with the retirement policy in China [30,31]. However, in geriatrics, the definition for older adults is age ≥65 years [32]. In cardiovascular medicine, “older” was defined as age ≥75 years [33]. Therefore, to better clarify the association between family health and frailty in “true” older adults, we performed subgroup analysis in different age groups, including “≥65 years old” and “≥75 years old.”

### Ethics Approval and Informed Consent

This quantitative study was performed in accordance with the guideline “involves people of biomedical research ethics review method (try out)” of the China Ministry of Health national drug supervision and administration of the quality control standard for clinical trials (2003), medical instrument clinical trial regulations (2004), and the Declaration of Helsinki. The investigators obtained ethics approval from the Shaanxi Institute of International Trade and Commerce (JKWH-2022-02). All applicable institutional and governmental regulations concerning the ethical use of human volunteers were followed over the course of this study. All interviewees provided written informed consent to participate in this study upon recruitment, and they participated voluntarily without compensation. All the study data were anonymous.

## Results

### Demographic Characteristics

Among the 3758 participants, 2327 (61.92%) were robust, 1342 (35.71%) were prefrail, and 89 (2.37%) were frail. The mean score of family health was 37.86 (SD 6.44), with 39.45 (SD 6.05), 35.18 (SD 6.25), and 36.63 (SD 5.53) in the robust, prefrail, and frail groups, respectively. We further compared the mean scores of family health between the prefrail and frail groups, and the difference was not significant (*P*=.237). Participants in the frail group were older than those in the other groups. The ratios for illiteracy; divorced, widowed, or unmarried; and smoking were also higher in the frail group than in the other groups, whereas self-rated health, health literacy, and the ratio of usually exercising (≥150 min/wk) were lower than those of the other groups (Table 2).

**Table 2:** Demographic characteristics and family health levels for participants aged ≥60 years by frailty status.

		All (N=3758)	Robust (n=2327)	Prefrail (n=1342)	Frail (n=89)	*P* value	
Age (years), mean (SD)		68.81 (6.26)	68.28 (6.03)	69.37 (6.40)	74.33 (6.92)	<.001	
**Sex, n (%)**							.51
	Male	1866 (49.65)	1173 (50.41)	650 (48.44)	43 (48.31)		
	Female	1892 (50.35)	1154 (49.59)	692 (51.56)	46 (51.69)		
**Family type^a^, n (%)**							<.001
	Core family	336 (8.94)	190 (8.17)	139 (10.36)	7 (7.86)		
	Backbone family	1876 (49.92)	1228 (52.77)	612 (45.6)	36 (40.45)		
	Joint family	237 (6.31)	138 (5.93)	92 (6.86)	7 (7.86)		
	Conjugal family	897 (23.87)	587 (25.23)	286 (21.31)	24 (26.97)		
	Single-parent family	143 (3.81)	67 (2.88)	70 (5.22)	6 (6.74)		
	Other	269 (7.16)	117 (5.03)	143 (10.66)	9 (10.11)		
**Residence, n (%)**							<.001
	Urban	2110 (56.15)	1408 (60.51)	664 (49.48)	38 (42.69)		
	Rural	1648 (43.85)	919 (39.49)	678 (50.52)	51 (57.3)		
**Marital status, n (%)**							<.001
	Married	3200 (85.15)	2034 (87.41)	1102 (82.12)	64 (71.91)		
	Divorced, widowed, or unmarried	558 (14.85)	293 (12.59)	240 (17.88)	25 (28.09)		
**Number of children, n (%)**							<.001
	0	277 (7.37)	96 (4.13)	175 (13.04)	6 (6.74)		
	1	1212 (32.25)	780 (33.52)	420 (31.3)	12 (13.48)		
	2	1232 (32.78)	804 (34.55)	402 (29.96)	26 (29.21)		
	≥3	1037 (27.59)	647 (27.8)	345 (25.71)	45 (50.56)		
**Public insurance coverage, n (%)**							<.001
	No	297 (7.9)	113 (4.86)	180 (13.41)	4 (4.49)		
	Yes	3461 (92.1)	2214 (95.14)	1162 (86.59)	85 (95.51)		
BMI (kg/m^2^), mean (SD)		21.91 (3.61)	22.10 (3.57)	21.62 (3.67)	21.38 (3.52)	<.001	
Self-rated health, mean (SD)		70.08 (18.35)	73.92 (16.99)	64.41 (18.42)	55.10 (21.60)	<.001	
Health literacy, mean (SD)		25.04 (5.29)	26.05 (4.96)	23.47 (5.37)	22.09 (5.41)	<.001	
**Physical exercise (minutes per week), n (%)**							<.001
	<150	557 (14.82)	275 (11.82)	246 (18.33)	36 (40.45)		
	≥150	3201 (85.18)	2052 (88.18)	1096 (81.67)	53 (59.55)		
**Alcohol intake, n (%)**							.18
	No	3237 (86.14)	2019 (86.76)	1144 (85.25)	74 (83.15)		
	Yes	521 (13.86)	308 (13.24)	198 (14.75)	15 (16.85)		
**Smoking status, n (%)**							<.001
	No	3125 (83.16)	2019 (86.76)	1040 (77.5)	66 (74.16)		
	Yes	633 (16.84)	308 (13.24)	302 (22.5)	23 (25.84)		
**Sleep duration per night (hours), n (%)**							<.001
	<5	255 (6.79)	88 (3.78)	152 (11.33)	15 (16.85)		
	5-6	801 (21.31)	389 (16.72)	391 (29.14)	21 (23.6)		
	6-7	1271 (33.82)	794 (34.12)	453 (33.76)	24 (26.97)		
	>7	1431 (38.08)	1056 (45.38)	346 (25.78)	29 (32.58)		
**Have breakfast every day, n (%)**							<.001
	No	939 (24.99)	381 (16.37)	529 (39.42)	29 (32.58)		
	Yes	2819 (75.01)	1946 (83.63)	813 (60.58)	60 (67.41)		
Family health, mean (SD)		37.86 (6.44)	39.45 (6.05)	35.18 (6.25)	36.63 (5.53)	<.001	

^a^“Core family” refers to a family consisting of parents and unmarried children; “Conjugal family” refers to a family consisting of parents and married children; “Backbone family” refers to a family consisting of only husband and wife; “Joint family” means a family of parents or more married children or siblings after marriage; “Single-parent family” means a family consisting of divorced, widowed, or unmarried single fathers or mothers and their children or adopted children; and “Other” consists of the following: “Intergenerational family,” referring to a family with only 2 generations, and the parents left the family for some reasons; “Dink family,” referring to a voluntary infertile family consisting of 2 couples; “Single family,” referring to not being married at the age of marriage or not married after divorce but living alone; and reformed families, cohabitation families, and gay families.

### The Association Between Family Health and Frailty

The ordered logistic regression (Table 3) showed that better family health was associated with a lower risk of frailty (OR 0.93, 95% CI 0.91-0.94), with covariates adjusted (model 1). After potential mediators were entered into the model (model 2), the association between family health and frailty was still significant (OR 0.94, 95% CI 0.93-0.96; *P*<.001). Model 2 had a greater pseudo *R*^2^ and lower Akaike information criterion and Bayesian information criterion, which indicated better model fitting performance. Meanwhile, except for no alcohol intake (*P*=.74), high health literacy (*P*=.01), not smoking (*P*=.009), physical exercise for ≥150 minutes per week (*P*<.001), more than 5 hours of sleep (all *P*<.05), and having breakfast every day (*P*<.001) were significantly associated with a decreased risk of frailty.

**Figure 2:**
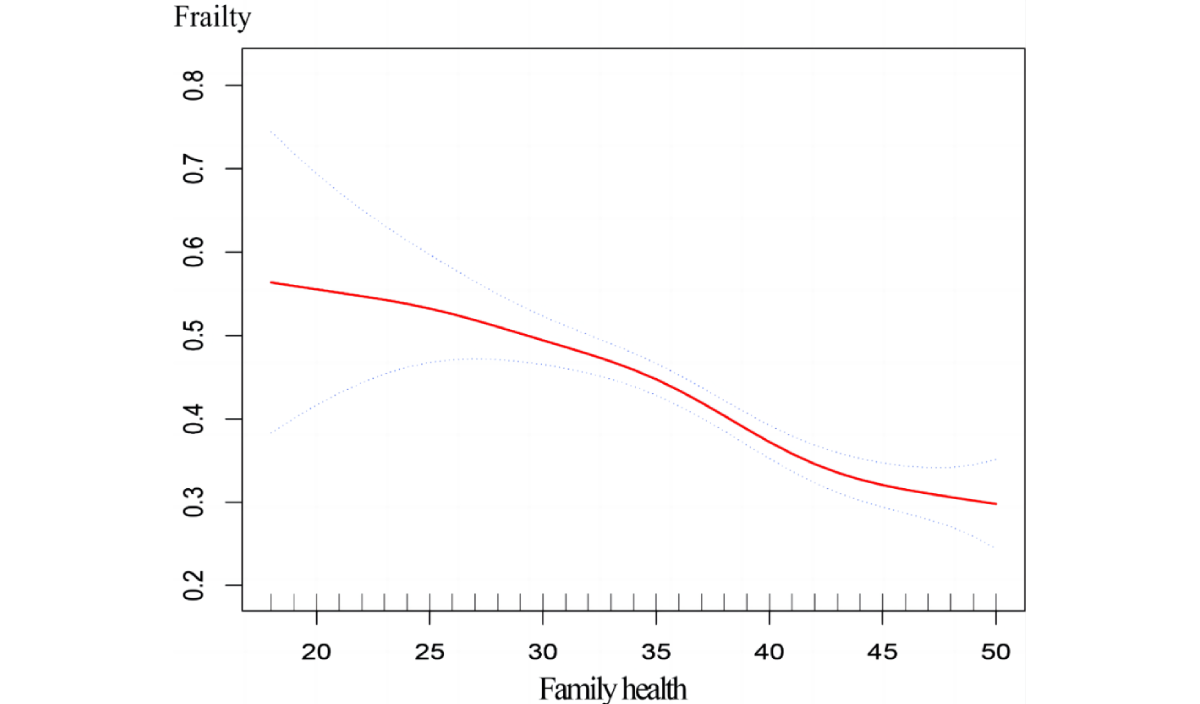
Smooth curve fitting for the association between family health and frailty, with all covariates and potential mediators controlled.

After adjusting for covariates and potential mediators, the smooth curve based on the generalized additive model suggested that family health tended to be linearly associated with frailty risk, and frailty risk decreased with increased family health (Figure 2).

We further analyzed the associations between the 4 dimensions of family health and frailty; the increases in all dimensions were associated with decreased frailty risk (Multimedia Appendix 3).

**Table 3:** The association between family health and frailty for participants aged ≥60 years explored by ordered logistic regression.

		Model 1			Model 2	
		OR^a^ (95% CI)	*P* value	OR (95% CI)		*P* value
Family health		0.93 (0.91-0.94)	<.001	0.94 (0.93-0.96)		<.001
Age		1.05 (1.04-1.07)	<.001	1.05 (1.04-1.06)		<.001
Sex (ref^b^: male)		1.13 (0.98-1.31)	.08	1.15 (0.99-1.35)		.07
BMI		0.99 (0.97-1.01)	.22	0.99 (0.97-1.01)		.45
**Family type (ref: core family)**						
	Backbone family	0.70 (0.54-0.90)	.005	0.65 (0.5-0.84)		.001
	Joint family	1.08 (0.75-1.56)	.67	0.95 (0.66-1.37)		.79
	Conjugal family	0.61 (0.46-0.81)	.001	0.59 (0.44-0.79)		<.001
	Single-parent family	1.01 (0.63-1.60)	.98	0.88 (0.55-1.42)		.60
	Other	0.94 (0.65-1.36)	.74	0.84 (0.57-1.22)		.36
Residence (ref: urban)		1.31 (1.13-1.52)	<.001	1.24 (1.06-1.45)		.006
Marital status (ref: married)		1.06 (0.83-1.34)	.65	1.05 (0.82-1.34)		.70
**Number of children (ref: 0)**						
	1	0.70 (0.52-0.96)	.03	0.95 (0.69-1.32)		.78
	2	0.66 (0.48-0.90)	.009	0.98 (0.7-1.37)		.92
	≥3	0.59 (0.43-0.82)	.001	0.86 (0.61-1.22)		.41
Self-rated health		0.98 (0.97-0.98)	<.001	0.98 (0.98-0.98)		<.001
Public insurance coverage (ref: not covered)		0.53 (0.41-0.69)	<.001	0.6 (0.46-0.78)		<.001
Health literacy		N/A^c^	N/A	0.98 (0.96-0.99)		.01
Smoking status (ref: smoke)		N/A	N/A	0.76 (0.61-0.93)		.009
Alcohol intake (ref: drink)		N/A	N/A	1.04 (0.83-1.30)		.74
Physical exercise (ref: <150 min/wk)		N/A	N/A	0.57 (0.47-0.70)		<.001
**Sleep duration per night (ref: <5 hours)**						
	5-6 hours	N/A	N/A	0.64 (0.48-0.87)		.004
	6-7 hours	N/A	N/A	0.42 (0.31-0.56)		<.001
	>7 hours	N/A	N/A	0.31 (0.23-0.41)		<.001
Have breakfast every day (ref: no)		N/A	N/A	0.63 (0.52-0.75)		<.001
Pseudo *R*^2^		0.121	N/A	0.16		N/A
Akaike information criterion		5013.02	N/A	4833.66		N/A
Bayesian information criterion		5125.19	N/A	4995.69		N/A

^a^OR: odds ratio.

^b^ref: reference.

^c^N/A: not applicable.

### Mediation Analysis

**Figure 3:**
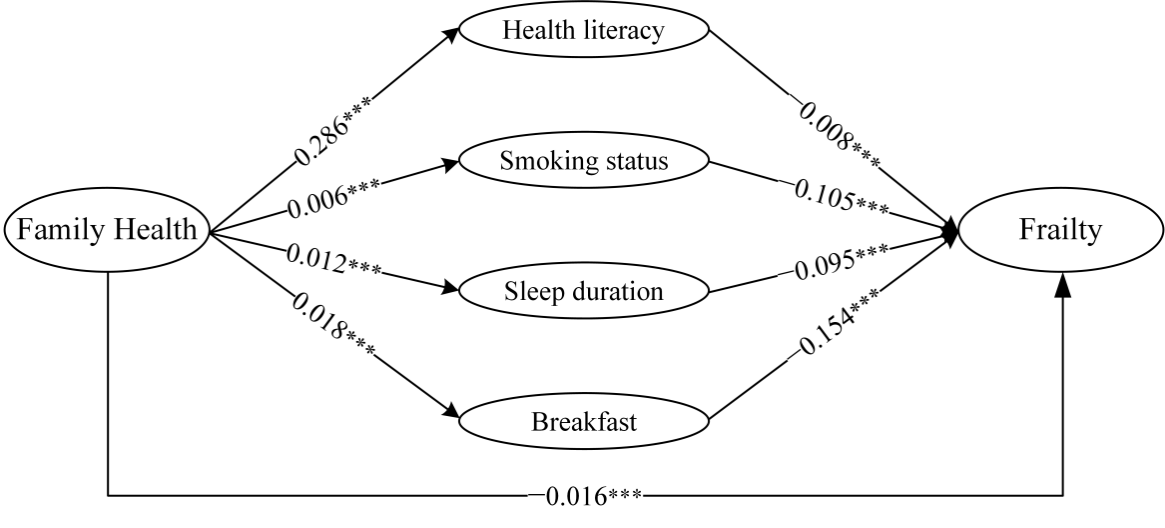
Mediation analysis of the association between family health and frailty. ***Indicates that the statistically significant association at α=.001 level.

Mediation analysis revealed that the total effect of family health on frailty was −0.016, and the association between family health and frailty was mediated by health literacy, not smoking, sleep duration, and having breakfast every day. The Sobel test results were significant (*P*<.001). The KHB composition revealed that the 4 mediators reduced the total effect of family health on frailty by 26.72%. In addition, the mediating effects of health literacy and having breakfast constituted 8.04% and 10.98%, respectively, which were much higher than those of the other 3 mediators. Further details are presented in Figure 3 and Table 4.

We explored the mediating effects of health literacy, not smoking, sleep duration, and having breakfast on the 4 dimensions of family health and frailty (Table 5). The results were similar, although the mediating effect of sleep duration on the association between family health resources and frailty was not significant (*P*=.354).

**Table 4:** The mediating effect of health literacy and health behaviors on family health and frailty explored by Sobel tests and Karlson-Holm-Breen (KHB) decomposition methods.

			Health literacy		Smoking status		Alcohol intake		Physical exercise		Sleep duration		Breakfast
Family health→mediator, β			.286^a^		.006^a^		.001		−.002		.012^a^		.018^a^
Mediator→frailty, β			−.008^a^		−.105^a^		−.023		−.138^a^		−.095^a^		−.154^a^
Indirect effect, β			−.002^a^		−.001^a^		−.000		.000		−.001^a^		−.003^a^
Direct effect, β			−.014^a^		−.016^a^		−.016^a^		−.017^a^		−.015^a^		−.014^a^
Total effect, β			−.016^a^		−.016^a^		−.016^a^		−.016^a^		−.016^a^		−.016^a^
Proportion of total effect that is mediated			0.147		0.042		0.001		−0.016		0.070		0.166
Sobel test			−0.002^a^		−0.001^a^		−0.000		−0.000		−0.001^a^		−0.003^a^
**KHB** **decomposition**													
	Proportion of mediation effect (%)	8.04		1.96		—^b^		—		5.74		10.98	

^a^*P*<.001.

^b^Alcohol intake and physical exercise were not taken into KHB analysis as the mediation effects were not significant.

**Table 5:** Mediation analysis and Karlson-Holm-Breen (KHB) decomposition of health literacy and health behaviors mediating the associations between dimensions of family health and frailty.

				Health literacy		Smoking status		Sleep duration		Breakfast
**Family, social, or emotional health processes**										
	Indirect effect, β		−.007^a^		−.002^a^		−.003^a^		−.007^a^	
	Direct effect, β		−.029^a^		−.034^a^		−.034^a^		−.030^a^	
	Total effect, β		−.037^a^		−.037^a^		−.037^a^		−.037^a^	
	**KHB decomposition**									
		Proportion of mediation effect (%)	10.78		2.89		6.20		13.23	
**Family healthy lifestyle**										
	Indirect effect, β		−.012^a^		−.004^a^		−.004^a^		−.010^a^	
	Direct effect, β		−.032^a^		−.041^a^		−.040^a^		−.034^a^	
	Total effect, β		−.044^a^		−.044^a^		−.044^a^		−.044^a^	
	**KHB decomposition**									
		Proportion of mediation effect (%)	16.00		3.67		7.86		16.31	
**Family health resources**										
	Indirect effect, β		.003^a^		.001^b^		−.000		−.002^a^	
	Direct effect, β		−.019^a^		−.017^a^		−.015^a^		−.014^a^	
	Total effect, β		−.016^a^		−.016^a^		−.016^a^		−.016^a^	
	**KHB decomposition**									
		Proportion of mediation effect (%)	−13.33		−3.99		2.47		7.57	
**Family external social supports**										
	Indirect effect, β		−.012^a^		−.003^a^		−.003^a^		−.008^a^	
	Direct effect, β		−.026^a^		−.035^a^		−.035^a^		−.030^a^	
	Total effect, β		−.038^a^		−.038^a^		−.038^a^		−.038^a^	
	**KHB decomposition**									
		Proportion of mediation effect (%)	17.23		3.67		6.63		14.01	

^a^*P*<.001.

^b^*P*<.01.

### Sensitivity Analysis

In sensitivity analysis (Multimedia Appendix 4), with deficits of frailty (scoring from 0 to 5) set as the dependent variable, KHB methods were used, and a negative abnormal regression model was used. The results were consistent with those of the main analysis. When using walking (at least 10 minutes) days per week to replace physical exercise time, the mediating effect was significant (*P*<.001), and the path to family health was positively associated with physical exercise and further decreased frailty risk. When using self-rated sleep quality to replace sleep duration, the results revealed that better family health was associated with better sleep quality and further decreased frailty risk, with a significant mediating effect (*P*<.001).

### Subgroup Analysis

To better clarify the association between family health and frailty in “true” older adults, we performed subgroup analyses in different age groups, including “≥65 years old” and “≥75 years old.” First, through ordered logistic regressions, the negative associations between family health and frailty were significant in the 2 subgroups (for those aged ≥65 years: OR 0.94, 95% CI 0.93-0.96, *P*<.001; for those aged ≥75 years: OR 0.96, 95% CI 0.93-0.99, *P*=.007). For those aged ≥65 years, the mediating effects of health literacy, smoking status, sleep duration, and breakfast remained significant. However, for those aged ≥75 years, only the mediating effect of breakfast was significant (for health literacy: *P*=.554; for smoking status: *P*=.266; for sleep duration: *P*=.081). The details are presented in Table 6.

**Table 6:** Table6. Subgroup mediation analysis stratified by age based on Sobel tests and Karlson-Holm-Breen (KHB) decomposition methods.

			Health literacy	Smoking status	Sleep duration	Breakfast
≥**65 years**						
	Indirect effect, β		−.002^a^	−.001^a^	−.001^b^	−.002^b^
	Direct effect, β		−.015^b^	−.016^b^	−.016^b^	−.014^b^
	Total effect, β		−.017^b^	−.017^b^	−.017^b^	−.017^b^
	**KHB decomposition**					
		Proportion of mediation effect (%)	4.87	1.33	5.92	9.30
≥**75 years**						
	Indirect effect, β		−.001	−.000	−.001	−.003^a^
	Direct effect, β		−.014^b^	−.014^b^	−.013^b^	−.012^a^
	Total effect, β		−.014^b^	−.014^b^	−.014^b^	−.014^b^
	**KHB decomposition**					
		Proportion of mediation effect (%)	—^c^	—	—	18.48

^a^*P*<.01.

^b^*P*<.001.

^c^Variables did not taken into KHB decomposition analysis as their mediating effects were not significant.

## Discussion

### Principal Findings

To our knowledge, this is the first study to assess and divide the effects of family health on frailty. The results demonstrate that family health is negatively associated with the prevalence of prefrailty and frailty, and this association can be mediated by health literacy and certain health behaviors (eg, not smoking, longer sleep duration, and having breakfast every day).

The prevalence of prefrailty and frailty in this study was 35.71% and 2.37%, respectively, which was lower than that reported in many previous studies in China. Furthermore, family health did not vary significantly between the prefrail and frail groups. This may be because the main survey method was conducted based on face-to-face reporting through an electronic questionnaire. If a respondent was mentally alert but not active enough to answer the questionnaire, the investigator conducted a one-on-one inquiry to complete the questionnaire on their behalf. Most participants were relatively healthy and less vulnerable than frail older adults. Therefore, the representativeness of the study sample might have been limited. In addition, frailty was measured using the FRAIL scale, which could not be validated by other frailty measures in this study, such as the frailty index proposed by Rockwood et al [34]. However, the findings of this study are important. Prefrail older adults are at a higher risk than robust older adults in experiencing frailty, adverse outcomes, and mortality [8]. In addition, older people with prefrailty are more likely to transition back to a state of robust health than those who are frail [35]. Therefore, health promotion for prefrail populations represents an important opportunity to prevent decline and dependence, enhance health, and reduce disability and the need for care. Therefore, the findings of this study provide important intervention strategies to manage frailty and have substantial implications for health policy and public health. Moreover, the results of the smooth curve and generalized additive model indicated a decreased risk of frailty and increased family health. Previous studies have indicated that both prefrail and frail older adults are highly susceptible to adverse health effects such as falls, disability, institutionalization, and hospitalization, all of which increase the social burden caused by population aging [36]. Therefore, improvement in family health is effective in preventing or delaying the onset of prefrailty or frailty.

Most studies associated with frailty management concentrated more on individuals, specifically exercise, nutritional intervention, multicomponent interventions, and individually tailored geriatric care models [37]. However, most individuals cannot exist away from their family, and family members are dependent on each other. Therefore, older adults may be far more affected by their family than by the outside world. As described by the family system theory, the family is a cohesive social unit that operates like a system with its own rules and responsibilities. Each family member has a profound impact on the choices of other members of the family, with the results passed on from generation to generation [38]. Therefore, frailty intervention in family units has great potential and may achieve twice the results with half the effort. This also indicates that promoting healthy aging through family health is effective and promising.

Previous studies have shown that the role of family is essential in predicting better health-related quality of life among older adults [39]. The presence of the family acts as the main source of social support in the acceptance of the aging process as well as helping to motivate participation in daily activities and improving self-esteem [40]. Psychosocial factors, such as individual preferences and values concerning food and sports or physical activities, affect health-related family interactions. Sociocultural factors indirectly affect family interactions via individuals who transfer these influences into their family lives [41]. In addition, the mediators in this study, including health literacy, smoking, sleep, and diet, have been shown to be effective in frailty interventions [42-44]. Family health, which covers a wider dimension of health-related social and internal support, interactions, and resources than family function or family climate [45], contributes to higher levels of health literacy and healthier behaviors and thus can decrease the risk of prefrailty and frailty. It is worth noting that in this study, the KHB composition revealed that the 4 mediators (health literacy, not smoking, sleep duration, and having breakfast every day) reduced the total effect of family health on frailty by only 26.72%, indicating that the proportion of mediated effects is not high. Family health is a comprehensive concept associated with individual’s health in many aspects. The frailty status is also complex and is affected by many risk factors. Therefore, this study indicated potential paths for addressing the association between family health and frailty. Other mechanisms, especially the clinical, psychological, or physiological paths, should be explored in future research.

It is intriguing that the mediating effect of physical exercise on the association between family health and frailty was not significant when measured by weekly time ≥150 minutes but significant when measured by days of walking for at least 10 minutes per week. First, the negative association between physical exercise and frailty risk was significant in this study, which has also been supported by many previous studies [46-48]. Second, physical exercise, especially intensive and initiative exercises, was more popular among younger adults than older adults in China, and these exercise behaviors may be not easy to form among older adults, even when encouraged or urged by their family members. However, this study still indicated that some easy exercises, such as walking, which are also associated with decreased frailty risk [49], could be promoted by improving family health.

Family health resources, one of the dimensions of frailty, are negatively associated with health literacy and not smoking and also negatively associated with frailty. This may be because health sources mainly contribute to other behaviors, such as health-seeking behavior. Doctors’ attempts to improve patients’ health literacy and motivate them to quit smoking may be inadequate.

In addition, this study indicated that family health was associated with decreased frailty risk in older adults, which was validated in older adults aged ≥60 years, ≥65 years, and ≥75 years. However, the mediating roles of health literacy and health behaviors were not consistent. Most mediating effects in this study were not significant, particularly for those aged ≥75 years. A possible explanation may be found in the individual heterogeneity, complexity of disease conditions, and greater vulnerability to physical and psychological status for the relatively older populations [50]. Therefore, intervention in frailty status is more difficult in relatively older populations, and the associations between family health and frailty may be more significantly mediated by factors related to disease control and treatment. This also indicates that the mechanisms of the impact of family health on frailty are complex and require further exploration through prospective analyses within different age groups.

To improve family health, we suggest that it is necessary to broaden the previous perspective of family structure and family function, enhance the role of family health, make full use of and strengthen the internal relationships of the family, enhance the external support of the family, improve the family social network, and carry out health management of the older population with the family as the unit. Especially in China, with the acceleration of urbanization and the deepening of population aging, increasingly complex family structures and a broader range of social determinants of health have raised many challenges for health strategies. A family-centered healthy aging promotion strategy is feasible, but there is a need to consider the family’s internal and social characteristics and to develop more scientific health promotion and management strategies suited to local conditions with the development of medical technology. Future research should further explore intervention strategies for family health and the causal effects of family health on individual health to promote healthy aging.

### Limitations

This study has a few limitations. First, some participants were excluded from the data analysis because of missing data or logical errors. We compared our data with the population sample survey data from the National Bureau of Statistics (NBS) in China and found that the constituting ratio of the population aged ≥65 years among those aged ≥60 years (62.77%) was lower than that of NBS data (75.01%) and that the sex ratio (female or male) in this study was 0.99, which was slightly higher than that of NBS data (0.93). In addition, people who were confused, experienced mental health difficulties, or had cognitive impairments were not enrolled in the survey. However, mental or cognitive deficits are also risk factors for frailty. Therefore, the representativeness of the study findings should be interpreted with caution. Second, as mentioned earlier, the sample size of the frail population was small, making it difficult to conduct further analyses of the mechanism by which family health affects frailty. Third, all variables were self-reported, and some health-related variables may not be accurate because of recall bias. Fourth, this study had the inherent limitations of a cross-sectional study. Fifth, health behaviors that may be associated with frailty were not comprehensively or precisely determined in this survey. For example, the frequency of different physical exercises, nutritional intake, and self-adjustment of mood should be further explored. However, the association between family health and a decreased risk of prefrailty and frailty was consistently found across multiple models and subgroups, which offers important hints and inspiration for future studies. Longitudinal and prospective analyses of the causal effect of family health on frailty and its underlying mechanisms should be conducted in future studies.

### Conclusions

In conclusion, family health can be an important intervention target that appears to be negatively linked to frailty in Chinese older adults. The mediation roles played by health literacy and health behaviors suggest that they can be effective in improving family health to promote healthier lifestyles as well as improving health literacy to delay, manage, and reverse frailty. The analysis related to the dimensions of family health may explain the mechanisms of the associations between family health, health literacy or behaviors, and frailty and guide future interventions. Strategies to intervene in frailty through family health in healthy aging and national public health strategies deserve more attention in the future.

## Appendix

Multimedia Appendix 1 Fried frailty phenotype and measurement.

Multimedia Appendix 2 Health literacy and Assignment Criteria.

Multimedia Appendix 3 The associations between dimensions of family health and frailty based on ordered logistic regression.

Multimedia Appendix 4 Sensitivity analysis.

## Abbreviations

FRAIL: Fatigue, Resistance, Ambulation, Illnesses, and Loss of weight
KHB: Karlson-Holm-Breen
NBS: National Bureau of Statistics
OR: odds ratio

## Data Availability

The data sets generated and analyzed during this study are not publicly available because the data still need to be used for other research but are available from the corresponding author on reasonable request.
