# Supplementary material for: The Association Between Family Health and Frailty With the Mediation Role of Health Literacy and Health Behavior Among Older Adults in China: Nationwide Cross-Sectional Study
Source: JMIR Public Health Surveill. 2023 Jun 27;9:e44486. doi: 10.2196/44486 (PMC10337466; doi:10.2196/44486)
Supplement: Multimedia Appendix 1 [file publichealth_v9i1e44486_app1.docx]

**Multimedia Appendix 1.** Fried frailty phenotype and measurement

| **Indicator** | **Measurement** | **Classifications** | **Prevalence in the sample** |
| --- | --- | --- | --- |
| Fatigue | Did you get tired most of last week? | 1=Yes; 0=No | 17.59% |
| Resistance | Can you go up a staircase? | 1=No; 0=Yes | 7.64% |
| Ambulation | Can you walk a block (500 meters) away? | 1=No; 0=Yes | 7.77% |
| Illness | Suffering from at least 5 chronic diseases:□ cataclasis  □ cataract  □ osteoporosis  □ arthritis  □ hypertension  □stroke (cerebral infarction, cerebral hemorrhage)  □ coronary disease  □ dyslipidemia  □ diabetes mellitus  □ therioma  □ benign tumour  □chronic respiratory disease (chronic obstructive pulmonary disease / asthma)  □ chronic renal disease  □ chronic stomach / enteritis  □ viral hepatitis (e. g.: hepatitis B)  □ fatty liver disease  □ Alzheimer's Disease  □ agitans paralysis  □mood disorders (anxiety disorders, depression, etc.)  □ Other chronic diseases | 1=Yes; 0=No | 2.13% |
| Loss of body weight | Have you lost more than 5% of your unexplained weight in the last 1 year? | 1=Yes; 0=No | 17.70% |
