# Supplementary material for: The Association Between Family Health and Frailty With the Mediation Role of Health Literacy and Health Behavior Among Older Adults in China: Nationwide Cross-Sectional Study
Source: JMIR Public Health Surveill. 2023 Jun 27;9:e44486. doi: 10.2196/44486 (PMC10337466; doi:10.2196/44486)
Supplement: Multimedia Appendix 2 [file publichealth_v9i1e44486_app2.docx]

**Multimedia Appendix 2.** Health literacy and Assignment Criteria

| 1.Can you find information on treatments of illnesses that concerning you? |
| --- |
| 2.Can you understand the instructions that come with your medicine? |
| 3.Can you call an ambulance in an emergency? |
| 4.Can you understand why you need health screenings (such as breast exam, blood sugar test, blood pressure)? |
| 5.Can you tell which vaccinations you may need?Can you find information on how to manage mental health problems like stress or depression? |
| 6.Can you decide how to protect yourself from illness based on advice from your family or friends? |
| 7.Do you think activities (such as meditation, exercise, walking, Pilates etc.) that are good for your mental well-being? |
| 8.Is it easy for you to understand information from media (such as Internet, newspaper, magazines) on how to get healthier? |
| 9.Join a sports club or exercise class if you want to? |

Each item was followed by 4 answers (Very difficult=1; Difficult=2; Easy=3; Very easy=4). Participants were asked to choose one answer after each item according to how easy or difficult the following acts are for them. The total score is the sum of each question, with a minimum score of 9 and a maximum score of 36. Higher scores indicate higher health literacy .
