# Supplementary material for: The Association Between Family Health and Frailty With the Mediation Role of Health Literacy and Health Behavior Among Older Adults in China: Nationwide Cross-Sectional Study
Source: JMIR Public Health Surveill. 2023 Jun 27;9:e44486. doi: 10.2196/44486 (PMC10337466; doi:10.2196/44486)
Supplement: Multimedia Appendix 3 [file publichealth_v9i1e44486_app3.docx]

**Multimedia Appendix 3.** The associations between dimensions of family health and frailty based on ordered logistic regression

|  | OR | 95%CI | P value |
| --- | --- | --- | --- |
| Family/social/emotional health processes | 0.89 | 0.86 to 0.92 | <.001 |
| Family healthy lifestyle | 0.89 | 0.85 to 0.94 | <.001 |
| Family health resources | 0.92 | 0.90 to 0.94 | <.001 |
| Family external social supports | 0.90 | 0.86 to 0.95 | <.001 |

Notes: OR, odds ratio; CI, Confidence Interval.
