# Supplementary material for: The Association Between Family Health and Frailty With the Mediation Role of Health Literacy and Health Behavior Among Older Adults in China: Nationwide Cross-Sectional Study
Source: JMIR Public Health Surveill. 2023 Jun 27;9:e44486. doi: 10.2196/44486 (PMC10337466; doi:10.2196/44486)
Supplement: Multimedia Appendix 4 [file publichealth_v9i1e44486_app4.docx]

**Multimedia Appendix 4.** Sensitivity analysis

|  | Health literacy | Smoking status | Physical exercise | Sleep duration | Breakfast |
| --- | --- | --- | --- | --- | --- |
| **KHB decomposition** |  |  |  |  |  |
| Proportion of mediation effect (%） | 7.65** | 0.75* | 5.08*** | 5.51*** | 7.01*** |
| **Using walking days to replace physical exercise** |  |  |  |  |  |
| Indirect effect | -0.002*** | -0.001*** | -0.001*** | -0.001*** | -0.003*** |
| Direct effect | -0.014*** | -0.016*** | -0.016*** | -0.015*** | -0.014*** |
| Total effect | -0.016*** | -0.016*** | -0.016*** | -0.016*** | -0.016*** |
| KHB decomposition |  |  |  |  |  |
| Proportion of mediation effect (%) | 8.08 | 2.13 | 2.21 | 5.70 | 10.07 |
| **Using sleep quality to replace sleep duration** |  |  |  |  |  |
| Indirect effect | -0.002*** | -0.001*** | -- | -0.001*** | -0.003*** |
| Direct effect | -0.014*** | -0.016*** | -- | -0.016*** | -0.014*** |
| Total effect | -0.016*** | -0.016*** | -- | -0.016*** | -0.016*** |
| KHB decomposition |  |  |  |  |  |
| Proportion of mediation effect (%) | 7.40 | 2.83 | -- | 3.85 | 11.84 |

Notes: **P*<.05; ***P*<.01; ****P*<.001. KHB, Karlson-Holm-Breen.
